# How campus restorative environments foster pro-environmental behavior among university students: the chain mediating roles of awe and nature connectedness

**DOI:** 10.3389/fpsyg.2026.1903437

**Published:** 2026-09-10

**Authors:** Zhuquan Yang, Yan Li

**Affiliations:** 1 University Engineering Research Center of Full-Chain Dynamic Innovation in Wellness Tourism, Hezhou University, Hezhou, China; 2 School of Tourism and Sport Health, Hezhou University, Hezhou, China

**Keywords:** awe, campus landscape scene experience, chain mediation, nature connectedness, pro-environmental behavior, university students

## Abstract

**Background:**

Cultivating young people’s pro-environmental behavior has become a critical concern for sustainable development. Campus landscapes, as natural-cultural composite environments that university students encounter regularly, may relate to their ecological emotions and environmental behavior in important ways. However, the underlying psychological mechanisms remain insufficiently understood.

**Objective:**

Drawing on Attention Restoration Theory and self-transcendent emotion theory, this study constructs and tests a chain mediation model in which campus landscape scene experience is associated with pro-environmental behavior sequentially through awe and nature connectedness.

**Methods:**

A questionnaire survey was conducted with 768 university students at Hezhou University, a national AAA-rated scenic campus in Guangxi, China, from September to December 2025. Measures comprised the Perceived Restorativeness Scale (PRS-11), the Awe Short Measure (Awe-SM), the Nature Relatedness scale (NR-6), and the Pro-environmental Behavior Scale (PEBS). Data were analyzed using SPSS 26.0 and AMOS 24.0 for descriptive statistics, common method bias tests, reliability and validity tests, structural equation modeling, and bootstrap mediation analysis, with gender, academic year, and frequency of nature contact included as control variables.

**Results:**

(1) Campus landscape scene experience was positively associated with pro-environmental behavior (*β* = 0.487, *p* < 0.001). (2) Awe mediated the association between campus landscape scene experience and pro-environmental behavior [indirect effect = 0.361, 95% CI = (0.290, 0.432)]. (3) Nature connectedness did not independently mediate this association [indirect effect = 0.005, 95% CI = (−0.002, 0.014)]. (4) The chain mediation through awe and then nature connectedness was significant [indirect effect = 0.040, 95% CI = (0.017, 0.068)]. These patterns remained stable after including control variables.

**Conclusion:**

Campus landscape scene experience is associated with university students’ pro-environmental behavior both directly and indirectly through an awe–nature connectedness chain pathway. The findings extend research on restorative environments and pro-environmental behavior and offer empirical guidance for ecological education and campus landscape design in higher education.

## 1 Introduction

Global environmental challenges such as climate change, biodiversity loss, and resource scarcity have made pro-environmental behavior (PEB) a central topic in environmental psychology and sustainability research (Stern, 2000; Larson et al., 2015). University students, who are in a formative period of value development and habit formation, are widely regarded as a key group for future ecological governance (Chang et al., 2026). Cultivating their environmental awareness and pro-environmental behavior matters not only for personal sustainable lifestyles but also for broader ecological civilization goals. Yet much existing research has focused on macro-level policy interventions, formal ecological education, or value-based persuasion, while the everyday natural-cultural environments that may shape university students’ pro-environmental behavior have received comparatively less attention.

Campus landscapes are natural environments that university students encounter routinely as part of daily campus life. They integrate natural elements such as woodlands, lakes, lawns, and horticultural areas with cultural elements such as museums, plazas, and landscape features, forming a natural-cultural composite restorative environment (Hartig et al., 2014; Xu et al., 2024). A growing body of research indicates that campus green and landscape spaces are associated with relief from academic stress, attention restoration, and positive emotional experience, mental health, and social cognition (Alves et al., 2022; Liu et al., 2024). For instance, Liu et al. (2024) reported that campus green space was linked to undergraduates’ restorative experience and place attachment, and Xu et al. (2024) found associations between campus green space, student well-being, and pro-environmental behavior. Nevertheless, evidence on how campus landscape scene experience relates to university students’ pro-environmental behavior—and through what intervening psychological processes—remains limited.

Attention Restoration Theory (ART) holds that environments characterized by being away, fascination, coherence, and compatibility help individuals recover from directed-attention fatigue and are associated with positive affect and open cognitive states (Kaplan, 1995; Pasini et al., 2014). Within restorative environments, individuals may not only replenish cognitive resources but also develop stronger emotional bonds with nature. Empirical work suggests that perceived environmental restorativeness is associated, directly or indirectly, with ecological behavior (Berto, 2014; Zhao et al., 2019; Zhang and Zhang, 2024). However, the affective and cognitive pathways that may link restorative experience and pro-environmental behavior remain incompletely specified, and few studies have examined these pathways in the everyday campus context (Lengieza and Swim, 2021).

Recent research has highlighted self-transcendent emotions, particularly awe, as a candidate emotional mechanism connecting nature experience with pro-social and pro-environmental behavior (Keltner and Haidt, 2003; Piff et al., 2015; Stellar et al., 2017). Awe is elicited when individuals encounter stimuli that are vast and that exceed their current cognitive frameworks, and it has been associated with a “small self” effect that may broaden concern for others, society, and the natural world (Bai et al., 2017; Yan et al., 2024). In parallel, connectedness to nature (CN)—a relatively stable cognitive-affective bond between humans and the natural world—is consistently among the strongest correlates of pro-environmental behavior (Mayer and Frantz, 2004; Capaldi et al., 2014). A small but growing literature has examined the awe–nature connectedness–pro-environmental behavior sequence (Yang et al., 2018; Ng et al., 2023; Liu et al., 2025). However, this work has largely focused on tourists, outdoor-recreation participants, or general adult samples (Yang et al., 2018; Yan et al., 2024), and few studies have systematically examined the integrated “environmental perception → self-transcendent emotion → nature connectedness → behavior” sequence in a routine campus landscape setting. This constitutes the specific gap the present study addresses.

Against this background, the present study draws on Attention Restoration Theory and self-transcendent emotion theory to construct and test a chain mediation model—campus landscape scene experience → awe → nature connectedness → pro-environmental behavior—using survey data from 768 university students. The study makes three contributions. First, it brings everyday campus environments into pro-environmental behavior research, addressing a gap concerning how routine nature contact relates to university students’ environmental behavior. Second, it integrates restorative perception, self-transcendent emotion, nature connectedness, and behavior within a single model, clarifying how an environment–emotion–cognition–behavior sequence may operate. Third, the findings offer practical implications for campus landscape design, ecological education, and sustainable campus development.

## 2 Theoretical background and hypotheses

The proposed model draws on two complementary theoretical frameworks whose integration is central to the present study. Attention Restoration Theory (ART) primarily explains the “front end” of the sequence: how the perceptual qualities of an environment enter an individual’s cognitive-affective system, restoring directed attention and creating an open, receptive psychological state (Kaplan, 1995; Hartig et al., 2014). ART, however, is less specific about the emotional and motivational processes that translate restorative perception into value-laden behavior such as environmental protection. Self-transcendent emotion theory addresses this “back end”: it specifies how a receptive state gives rise to self-transcendent emotions—above all awe—that diminish self-focus, expand the boundaries of the self, and strengthen identification with entities beyond the self, including the natural world (Keltner and Haidt, 2003; Stellar et al., 2017; Yaden et al., 2017). Read together, the two theories describe a coherent chain: a restorative environment (ART) provides the perceptual precondition for awe, and awe (self-transcendent emotion theory) provides the affective mechanism that deepens nature connectedness and, ultimately, motivates pro-environmental behavior. In this integrated account, neither theory alone is sufficient—ART explains why the campus landscape is perceptually engaging, while self-transcendent emotion theory explains why that engagement becomes behaviorally consequential. This logic underlies the hypotheses developed below.

### 2.1 Campus landscape scene experience and pro-environmental behavior

Pro-environmental behavior refers to actions taken by individuals to reduce environmental harm, support ecological protection, and promote sustainable development (Stern, 2000; Larson et al., 2015). Environmental psychology suggests that the environment is not merely a physical backdrop for behavior; it may also relate to behavioral tendencies through its associations with cognition, emotion, and values (Schultz, 2001). Attention Restoration Theory holds that environments with restorative qualities are associated with recovery from daily stress, replenishment of cognitive resources, and positive psychological experiences (Kaplan, 1995; Hartig et al., 1997). Campus landscapes, as natural environments encountered routinely by university students, possess restorative qualities that may relate to their attention to and identification with nature, and in turn to environmental awareness and pro-environmental behavior (Liu et al., 2024; Xu et al., 2026).

Prior studies have reported that natural environment experience is associated with environmental responsibility, environmental attitudes, and ecological protection behavior (Hartig et al., 2014; Zhao et al., 2019). Compared with individuals who have limited nature contact, those who engage more frequently with natural environments tend to report more positive ecological values and stronger resource conservation and environmental protection behaviors (Capaldi et al., 2014; Barrera-Hernández et al., 2020). Accordingly, a high-quality campus landscape experience may serve as an important contextual correlate of pro-environmental behavior among university students. We therefore propose:

*H1:* Campus landscape scene experience is positively associated with university students’ pro-environmental behavior.

### 2.2 The mediating role of awe

Awe is a prototypical self-transcendent emotion typically elicited when individuals encounter stimuli that are vast or that exceed prior cognitive experience (Keltner and Haidt, 2003). Keltner and Haidt (2003) characterize awe as combining perceived vastness and a need for accommodation. When immersed in natural environments, individuals often report awe in response to grandeur, complexity, and vitality (Shiota et al., 2007; Bethelmy and Corraliza, 2019).

Although the lakes, woodlands, tea fields, and horticultural areas on a university campus are limited in scale, their ecological richness and immersive qualities may still elicit awe in students (Piff et al., 2015; Yan et al., 2024). Piff et al. (2015) reported across several studies that awe is associated with attenuated self-focus, a “small self” perception, and a stronger sense of connection with the wider world (Stellar et al., 2017). A growing body of research further suggests that awe is associated with greater attention to ecological systems and environmental issues, and with a stronger inclination to engage in environmental protection (Yang et al., 2018; Liu et al., 2025).

Thus, campus landscape scene experience may relate to pro-environmental behavior in part through awe. We therefore propose:

*H2:* Campus landscape scene experience is positively associated with awe.

*H3:* Awe is positively associated with pro-environmental behavior.

*H4:* Awe mediates the association between campus landscape scene experience and pro-environmental behavior.

### 2.3 The mediating role of nature connectedness

Nature connectedness refers to the cognitive and affective sense of close relatedness between the self and the natural environment (Mayer and Frantz, 2004; Nisbet et al., 2009). Schultz proposed that when individuals incorporate nature into their self-concept (inclusion of nature in self), they more readily develop ecological responsibility and pro-environmental attitudes (Schultz, 2002). Tam’s systematic review reported that nature connectedness is among the more stable correlates of pro-environmental behavior (Tam, 2013; Lengieza and Swim, 2021).

Campus landscapes not only provide university students with opportunities for nature contact but may also create conditions for establishing affective bonds with the natural world (Alves et al., 2022). Through sustained engagement with campus natural environments, students may gradually develop a sense of identification with and belonging to nature (Nisbet and Zelenski, 2013). Empirical research indicates that nature connectedness is associated with a range of pro-environmental behaviors, including resource conservation, ecological protection, and environmental participation (Capaldi et al., 2014; Chang et al., 2026). Thus, campus landscape scene experience may relate to pro-environmental behavior in part through nature connectedness. We therefore propose:

*H5:* Campus landscape scene experience is positively associated with nature connectedness.

*H6:* Nature connectedness is positively associated with pro-environmental behavior.

*H7:* Nature connectedness mediates the association between campus landscape scene experience and pro-environmental behavior.

### 2.4 The chain mediating role of awe and nature connectedness

Self-transcendent emotion theory holds that awe may help individuals transcend self-centered perspectives and strengthen their sense of connection with the broader world (Yaden et al., 2017; Stellar et al., 2017). When awe is elicited in natural settings, individuals may become more attentive to human-nature relationships and more inclined to view themselves as part of a larger ecological system, a shift that may deepen nature connectedness (Zhang et al., 2014; Ng et al., 2023).

Studies have reported that awe is associated with pro-environmental behavior both directly and indirectly via nature connectedness (Yang et al., 2018; Liu et al., 2025). In this view, natural-environment experience may first elicit awe; awe may then strengthen the affective bond between the self and nature; and this strengthened nature connectedness may, in turn, relate to pro-environmental behavior. Awe and nature connectedness may therefore jointly constitute a psychological pathway linking campus landscape scene experience and pro-environmental behavior. Consistent with the integrated ART–self-transcendent-emotion logic outlined above, we propose:

*H8:* Awe is positively associated with nature connectedness.

*H9:* Awe and nature connectedness sequentially mediate the association between campus landscape scene experience and pro-environmental behavior.

**Figure 1:**
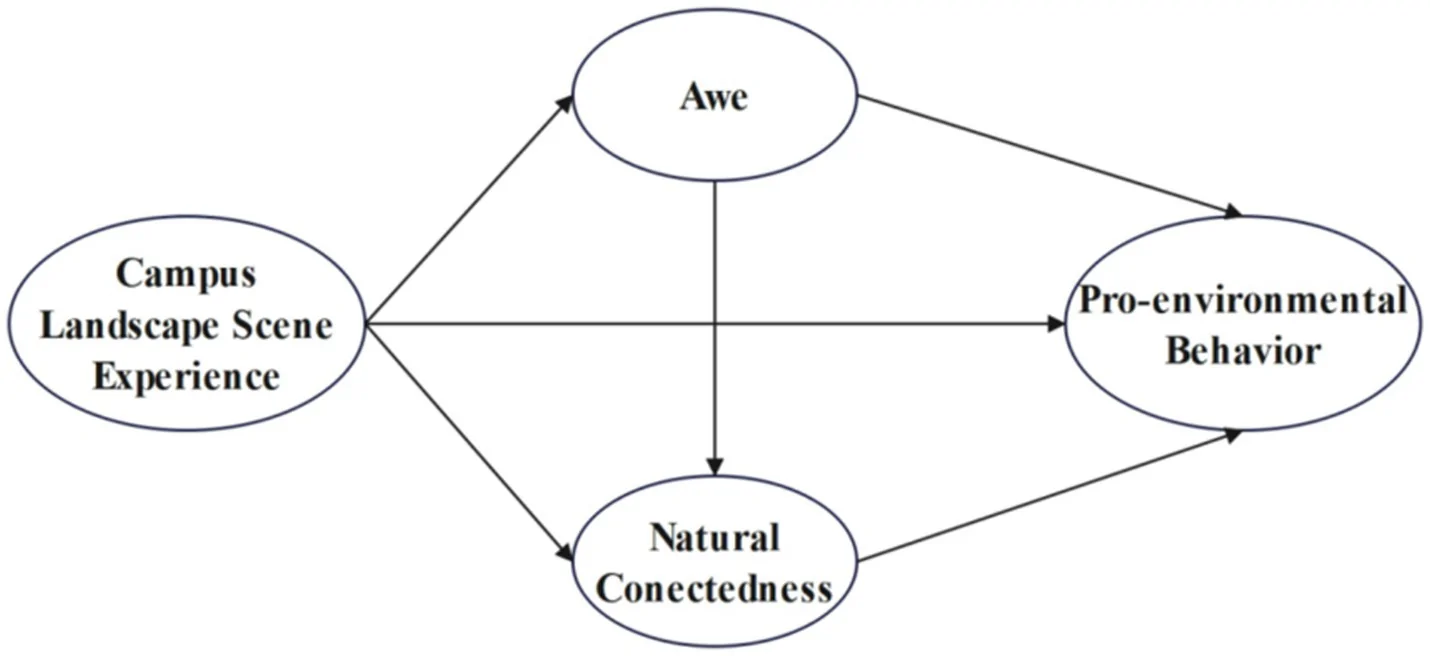
Conceptual model.

The theoretical model of this study is presented in Figure 1.

## 3 Methods

### 3.1 Variables and measures

This study examined four core variables—campus landscape scene experience, awe, nature connectedness, and pro-environmental behavior—within a chain mediation framework. All variables were measured using established scales widely used in international research. Items were rated on a five-point Likert scale ranging from 1 (“strongly disagree”) to 5 (“strongly agree”), with higher scores indicating higher levels of the corresponding construct.

1. *Campus landscape scene experience*. Campus landscape scene experience refers to students’ subjective perception of the restorative qualities of the natural and cultural landscapes on campus. It was measured using the short form of the Perceived Restorativeness Scale (PRS-11), originally developed by Hartig et al. (1997) on the basis of Attention Restoration Theory and revised by Pasini et al. (2014). The scale captures the dimensions of being away, fascination, coherence, and scope. Because the theoretical construct of interest is the campus landscape scene experience as a whole, the PRS-11 was used as its operational measure throughout.
2. *Awe.* Awe was conceptualized as a self-transcendent emotional experience elicited by stimuli that are vast or that exceed everyday experience. It was measured using the Awe Short Measure (Awe-SM) developed by Kannis-Dymand and colleagues (Kannis-Dymand et al., 2020), which comprises three dimensions—connectedness, absorption, and perceived vastness—with nine items in total.
3. *Nature connectedness.* Nature connectedness refers to the affective bond and identity overlap between the self and the natural environment. It was measured using the Nature Relatedness Short Form (NR-6) developed by Nisbet and Zelenski (2013), which consists of two dimensions—self and experience—with six items in total.
4. *Pro-environmental behavior.* Pro-environmental behavior refers to actions individuals take to protect the environment and promote sustainable development. It was measured using the Pro-environmental Behavior Scale (PEBS) developed by Larson and colleagues (Larson et al., 2015), which includes four dimensions—conservation lifestyle, land stewardship, social environmentalism, and environmental citizenship—with 13 items in total.
5. *Control variables.* Following recommendations in the pro-environmental behavior literature (Larson et al., 2015; Lengieza and Swim, 2021), gender, academic year, and frequency of contact with campus nature were included as control variables in the structural analyses to reduce the influence of demographic and exposure-related confounds on the estimated associations.

### 3.2 Study site, sample, and data collection

Data were collected at Hezhou University, located in Hezhou, Guangxi Zhuang Autonomous Region, China. The campus is a nationally designated AAA-rated scenic area, with abundant natural and cultural landscape resources including woodlands, tea fields, horticultural areas, lakes, and a folk culture museum. This setting provides students with routine opportunities for nature contact, leisure, and environmental cognition, making it a suitable and ecologically valid context for examining campus landscape scene experience. At the same time, the use of a single, landscape-rich campus defines the boundary of the sample and is revisited in the Limitations section.

The survey was conducted from September to December 2025. A combination of cluster and stratified sampling was used: strata were defined by academic year (first through fourth year), and questionnaires were then administered to intact classes selected within each stratum. This procedure was intended to improve the representativeness of the sample across academic years. Participants reported their academic major through an open-ended item rather than by selecting from predefined categories; the resulting sample spanned 55 distinct majors, broadly encompassing the humanities and social sciences, science and engineering, and arts and physical education. Because majors were recorded as free-text entries, participants were not assigned to predefined disciplinary strata, and the sample was not stratified by discipline.

Questionnaires were distributed and collected on site under the supervision of the research team, with a minimum recommended completion time of 15 min. Prior to administration, the research team explained the study purpose, completion instructions, and ethical considerations, emphasizing that responses would be used solely for academic research and would be fully anonymized. Participants responded on the basis of their actual experience of campus landscape spaces, including woodlands, lakes, tea fields, horticultural areas, and the museum.

A total of 900 questionnaires were distributed and 866 were returned (a return rate of 96.2%). Questionnaires completed in unrealistically short times, exhibiting obvious uniform-response patterns, or containing substantial missing data were excluded. After screening, 768 valid questionnaires were retained, yielding an effective response rate of 85.3%.

In the final sample, 308 participants (40.1%) were male and 460 (59.9%) were female. Participants ranged in age from 18 to 23 years, with those aged 19–21 accounting for 74.09% of the sample (18 years: *n* = 71, 9.24%; 19 years: *n* = 243, 31.64%; 20 years: *n* = 163, 21.22%; 21 years: *n* = 163, 21.22%; 22 years: *n* = 100, 13.02%; 23 years: *n* = 28, 3.65%). In terms of academic year, 246 (32.03%) were freshmen, 172 (22.40%) sophomores, 210 (27.34%) juniors, and 140 (18.23%) seniors. Regarding the frequency of outdoor nature contact per week, 205 participants (26.69%) reported one occasion, 189 (24.61%) two occasions, 198 (25.78%) three occasions, 49 (6.38%) four occasions, and 127 (16.54%) five or more occasions. This variable was subsequently included as a control in the structural analyses. Information on participants’ prior environmental coursework or environmental education was not collected in the present survey; this is acknowledged as a limitation in Section 5.4.

### 3.3 Ethical statement

This study was conducted in accordance with the principles of the Declaration of Helsinki. The research protocol was reviewed and approved by the Ethics Committee of the School of Tourism and Sports Health, Hezhou University (Approval No. HZUSTSH-0702), on July 1, 2025. Participation was entirely voluntary, and all participants provided written informed consent prior to completing the questionnaire. Participants were informed that they could withdraw at any time without consequence. No personally identifying information was collected, and all responses were anonymized and stored securely, used solely for the purposes of this academic research.

### 3.4 Data analysis

Data were analyzed using SPSS 26.0 and AMOS 24.0. First, SPSS 26.0 was used for descriptive statistics, common method bias testing, and reliability analysis using Cronbach’s alpha. Second, AMOS 24.0 was used to conduct confirmatory factor analysis (CFA) to examine convergent and discriminant validity of the measurement model, and to compute composite reliability (CR) and average variance extracted (AVE). Discriminant validity was assessed using both the Fornell–Larcker criterion and the heterotrait–monotrait ratio of correlations (HTMT).

Building on these analyses, structural equation modeling (SEM) was conducted to examine the associations among campus landscape scene experience, awe, nature connectedness, and pro-environmental behavior, controlling for gender, academic year, and frequency of nature contact. Model fit was evaluated using *χ*^2^/df, RMSEA, SRMR, CFI, and TLI (Hu and Bentler, 1999). Finally, bootstrap resampling with 5,000 replications was used to test the mediating roles of awe and nature connectedness and the chain mediating pathway. A mediating effect was considered statistically significant when the 95% confidence interval did not include zero (Hayes, 2018).

## 4 Results

### 4.1 Common method bias

Because all variables were measured using self-reported questionnaires collected from the same respondents, common method variance was assessed using two complementary statistical procedures. First, Harman’s single-factor test was conducted by entering all measurement items into an unrotated exploratory factor analysis (Podsakoff et al., 2003). The results yielded 11 factors with eigenvalues greater than 1, with the first factor accounting for 16.089% of the total variance. This proportion was substantially below the conventional 40% threshold, and no single factor accounted for the majority of the covariance among the measurement items.

Recognizing that Harman’s single-factor test alone provides only a preliminary assessment, an unmeasured latent method construct approach was additionally employed (Podsakoff et al., 2003). A latent common method factor was incorporated into the confirmatory measurement model, and each item was allowed to load on both its corresponding theoretical construct and the common method factor. The resulting model was then compared with the baseline measurement model. Although the inclusion of the common method factor produced a slight improvement in model fit, the magnitude of the change was limited (ΔCFI = 0.014). This modest improvement suggests that the common method factor provided little incremental explanatory value beyond the theoretically specified measurement structure.

Taken together, the results of the two complementary analyses indicate that common method variance may be present to some extent, as is possible in self-report research, but it is unlikely to have substantially distorted the measurement structure or the relationships examined in this study.

### 4.2 Exploratory factor analysis

To examine the structure of the scales, principal component analysis with varimax rotation was conducted on all measurement items. The Kaiser–Meyer–Olkin (KMO) measure was 0.978, well above 0.90, and Bartlett’s test of sphericity was significant (*χ*^2^ = 32336.908, df = 741, *p* < 0.001), indicating that the data were suitable for factor analysis (see Table 1).

**Table 1:** KMO and Bartlett’s test.

Test	Statistic	Value
Kaiser–Meyer–Olkin (KMO)	KMO	0.978
Bartlett’s test of sphericity	Approx. *χ*^2^	32336.908
	df	741
	Sig.	<0.001

Eleven factors with eigenvalues greater than 1 were extracted, cumulatively explaining 83.831% of the variance, well above the 60% recommended threshold. After rotation, all standardized loadings of items on their respective factors exceeded 0.50, with no substantial cross-loadings, indicating a sound factor structure.

### 4.3 Reliability and validity

Reliability and validity were assessed using Cronbach’s alpha, composite reliability (CR), average variance extracted (AVE), and confirmatory factor analysis (CFA). Results are presented in Table 2.

**Table 2:** Confirmatory factor analysis and reliability/validity test results.

Factor	Measured item	Unstd. loading (Coef.)	Std. error	*z* (CR)	Std. loading (estimate)	SMC	Cronbach’s *α*	C. R.	AVE
PRS	Fascination1	1	–	–	0.875	0.765	0.929	0.930	0.816
	Fascination2	1.074	0.030	35.602	0.897	0.804			
	Fascination3	1.115	0.029	38.859	0.938	0.879			
	Being Away1	1	–	–	0.829	0.687	0.880	0.883	0.716
	Being Away2	0.962	0.036	27.052	0.821	0.674			
	Being Away3	1.024	0.034	30.486	0.888	0.789			
	Coherence1	1	–	–	0.906	0.822	0.905	0.909	0.768
	Coherence2	1.029	0.027	38.074	0.898	0.806			
	Coherence3	0.950	0.030	31.611	0.824	0.679			
	Scope1	1	–	–	0.897	0.805	0.863	0.863	0.759
	Scope2	0.929	0.028	32.753	0.845	0.714			
PEB	Conservation Lifestyle1	1	–	–	0.844	0.712	0.887	0.887	0.725
	Conservation Lifestyle2	0.968	0.032	29.866	0.843	0.710			
	Conservation Lifestyle3	1.036	0.033	31.375	0.867	0.751			
	Land Stewardship1	1	–	–	0.847	0.717	0.867	0.866	0.683
	Land Stewardship2	0.997	0.034	29.725	0.832	0.692			
	Land Stewardship3	0.923	0.033	27.885	0.800	0.641			
	Social Environmentalism1	1	–	–	0.855	0.731	0.901	0.902	0.755
	Social Environmentalism2	0.987	0.030	33.071	0.881	0.776			
	Social Environmentalism3	0.985	0.030	32.415	0.871	0.759			
	Environmental Citizenship1	1	–	–	0.816	0.665	0.904	0.910	0.716
	Environmental Citizenship2	1.195	0.041	29.375	0.867	0.752			
	Environmental Citizenship3	1.199	0.039	30.524	0.889	0.790			
	Environmental Citizenship4	1.268	0.048	26.582	0.812	0.659			
AWE	Connectedness1	1	–	–	0.863	0.744	0.901	0.903	0.756
	Connectedness2	1.041	0.032	32.815	0.874	0.764			
	Connectedness3	1.111	0.034	32.605	0.871	0.759			
	Absorption1	1	–	–	0.562	0.316	0.765	0.799	0.577
	Absorption2	1.145	0.067	17.153	0.833	0.694			
	Absorption3	1.279	0.074	17.354	0.851	0.724			
	Perception1	1	–	–	0.919	0.844	0.911	0.912	0.776
	Perception2	0.981	0.026	37.456	0.873	0.762			
	Perception3	0.912	0.026	35.057	0.849	0.721			
CN	Self1	1	–	–	0.819	0.671	0.891	0.889	0.729
	Self2	1.071	0.037	28.882	0.860	0.740			
	Self3	1.081	0.036	29.981	0.881	0.776			
	Experience1	1	–	–	0.893	0.797	0.912	0.912	0.775
	Experience2	1.004	0.027	37.669	0.899	0.809			
	Experience3	0.936	0.028	33.052	0.847	0.718			

A dash (−) indicates the reference item. CR, composite reliability; AVE, average variance extracted; PRS, campus landscape scene experience (perceived restorativeness); AWE, awe; CN, nature connectedness; PEB, pro-environmental behavior.

Standardized factor loadings ranged from 0.562 to 0.938, all exceeding 0.50, indicating that the items adequately reflected their corresponding constructs. Cronbach’s alpha coefficients ranged from 0.765 to 0.929 and composite reliability (CR) values ranged from 0.799 to 0.930, all above the 0.70 threshold, supporting internal consistency and construct reliability. Average variance extracted (AVE) values ranged from 0.577 to 0.816, all exceeding 0.50, supporting convergent validity.

Discriminant validity was evaluated using the Fornell–Larcker criterion and the heterotrait–monotrait ratio of correlations. As shown in Table 3, the Fornell–Larcker criterion was satisfied for the construct pairs involving nature connectedness. However, the correlation between CLSE and awe was 0.813, marginally exceeding the square root of the AVE for CLSE (0.812). More substantial overlap was observed for PEB: its correlations with CLSE and awe were both 0.889, exceeding the square roots of the AVE for the corresponding constructs. Thus, the Fornell–Larcker results did not uniformly support discriminant validity across all construct pairs.

**Table 3:** Discriminant validity assessed using the Fornell–Larcker criterion.

Construct	CLSE	AW	CN	PEB
CLSE	**0.812**			
AW	0.813	**0.822**		
CN	0.608	0.722	**0.859**	
PEB	0.889	0.889	0.687	**0.824**

Bold diagonal values are the square roots of the AVE; off-diagonal values are inter-construct correlations.

The HTMT estimates, presented in Table 4, ranged from 0.641 to 0.931. The values for CLSE–CN, AW–CN, and CN–PEB were below the stricter criterion of 0.85. The HTMT value for CLSE–AW was 0.857, which was slightly above 0.85 but remained below 0.90. The values for CLSE–PEB and AW–PEB were 0.926 and 0.931, respectively, both exceeding the conventional 0.90 criterion and indicating substantial empirical overlap between PEB and its two antecedent constructs. The bootstrapped 95% confidence intervals for these estimates likewise fell largely above 0.90 [CLSE–PEB: (0.907, 0.944); AW–PEB: (0.907, 0.951)]. Although the upper bounds of all confidence intervals remained below 1.00, this does not by itself establish adequate discriminant validity. We therefore do not claim that these constructs are clearly distinct, and we conclude that discriminant validity is not established for the CLSE–PEB and AW–PEB pairs.

**Table 4:** Discriminant validity assessed using the heterotrait–monotrait ratio.

Construct pair	HTMT	95% CI lower	95% CI upper
CLSE–AW	0.857	0.825	0.885
CLSE–CN	0.641	0.575	0.698
CLSE–PEB	0.926	0.907	0.944
AW–CN	0.762	0.704	0.811
AW–PEB	0.931	0.907	0.951
CN–PEB	0.721	0.661	0.773

Additional evidence was obtained from alternative measurement-model comparisons. The hypothesized four-construct model provided a significantly better fit than models combining CLSE with awe, CLSE with PEB, or awe with PEB. Taken together, the evidence indicates that CLSE, awe, nature connectedness, and PEB are not empirically redundant, since collapsing any of these pairs significantly degrades model fit; at the same time, PEB shares a considerable portion of its variance with both CLSE and awe, and the four constructs cannot be treated as cleanly separable.

This overlap has direct implications for the interpretation of the structural results reported below. Because CLSE, awe, and PEB share substantial variance, the CLSE → PEB and AW → PEB coefficients, and the awe-mediated indirect effect in particular, should be read as upper-bound estimates that may partly reflect shared construct or common-method variance rather than fully distinct psychological processes. Accordingly, the direction and relative ordering of the pathways—notably the contrast between the significant AW → CN path and the non-significant CLSE → CN path, which would be unlikely to emerge if the constructs were interchangeable—provide a firmer basis for inference than the absolute magnitudes of the coefficients. This limitation is revisited in Section 5.4.

### 4.4 Measurement model assessment and alternative model comparison

Confirmatory factor analysis was conducted to assess the fit of the hypothesized measurement model. The four-construct model demonstrated an acceptable fit to the data, with *χ*^2^ = 3288.58, df = 683, *χ*^2^/df = 4.815, CFI = 0.919, TLI = 0.912, RMSEA = 0.071, and SRMR = 0.035. These indices were evaluated against established criteria in the structural equation modeling literature (Wheaton et al., 1977; Bentler, 1990; Browne and Cudeck, 1993; Hu and Bentler, 1999; Kline, 2016; Fornell and Larcker, 1981; Henseler et al., 2015). The *χ*^2^/df ratio falls within the threshold of <5 commonly cited following Wheaton et al. (1977), although it approaches the upper boundary of that range; more conservative recommendations propose a criterion of <3 (Kline, 2016). Because the *χ*^2^ statistic is known to be inflated in large samples, and the present sample comprises 768 respondents, the overall assessment of model fit rests primarily on indices less sensitive to sample size: RMSEA was below the 0.08 criterion for acceptable fit (Browne and Cudeck, 1993), SRMR was below the 0.08 threshold (Hu and Bentler, 1999), and the incremental fit indices CFI and TLI both exceeded 0.90 (Bentler, 1990; Hu and Bentler, 1999). Taken together, these indices indicate acceptable, though not excellent, model fit.

To examine whether the highly correlated constructs were more appropriately represented as separate or combined factors, the hypothesized model was compared with three alternative measurement models. As shown in Table 5, combining CLSE and awe resulted in a significant deterioration in model fit, Δ*χ*^2^(3) = 450.84, *p* < 0.001. Model fit also deteriorated significantly when CLSE and PEB were combined, Δ*χ*^2^(3) = 156.95, *p* < 0.001, and when awe and PEB were combined, Δ*χ*^2^(3) = 259.00, *p* < 0.001. These results supported the four-construct measurement structure.

**Table 5:** Alternative measurement model comparisons.

Model	*χ* ^2^	df	*χ*^2^/df	CFI	TLI	RMSEA	SRMR	Δ*χ*^2^	Δdf
Four-construct model	3288.58	683	4.815	0.919	0.912	0.071	0.035	—	—
CLSE and AW combined	3739.42	686	5.451	0.905	0.898	0.076	0.043	450.84***	3
CLSE and PEB combined	3445.53	686	5.023	0.914	0.907	0.072	0.038	156.95***	3
AW and PEB combined	3547.58	686	5.171	0.911	0.904	0.074	0.038	259.00***	3

***p < 0.001.

### 4.5 Structural model and hypothesis testing

Structural equation modeling was used to examine the associations among campus landscape scene experience, awe, nature connectedness, and pro-environmental behavior, with gender, academic year, and frequency of nature contact included as control variables. Results for the hypothesized paths are presented in Table 6.

**Table 6:** SEM path coefficients (*n* = 768, with control variables).

Path	*B*	SE	*t*	*p*	*β*	Decision
CLSE → PEB (H1)	0.491	0.022	22.102	< 0.001	0.487	Supported
CLSE → AW (H2)	0.804	0.021	38.592	< 0.001	0.813	Supported
AW → PEB (H3)	0.448	0.026	17.421	< 0.001	0.440	Supported
CLSE → CN (H5)	0.069	0.046	1.478	0.140	0.063	Not supported
CN → PEB (H6)	0.069	0.017	3.976	< 0.001	0.074	Supported
AW → CN (H8)	0.732	0.047	15.631	< 0.001	0.670	Supported

Coefficients are from the model including gender, academic year, and frequency of nature contact as controls; hypothesized-path estimates were substantively unchanged from the model without controls. CLSE, campus landscape scene experience; AW, awe; CN, nature connectedness; PEB, pro-environmental behavior.

Campus landscape scene experience was positively associated with awe (*β* = 0.813, *t* = 38.592, *p* < 0.001), supporting *H2*. Awe was positively associated with nature connectedness (*β* = 0.670, *t* = 15.631, *p* < 0.001), supporting *H8*. Nature connectedness was positively associated with pro-environmental behavior (*β* = 0.074, *t* = 3.976, *p* < 0.001), supporting *H6*. Campus landscape scene experience was positively associated with pro-environmental behavior (*β* = 0.487, *t* = 22.102, *p* < 0.001), and awe was likewise positively associated with pro-environmental behavior (*β* = 0.440, *t* = 17.421, *p* < 0.001), supporting *H1* and *H3*.

In contrast, the direct association between campus landscape scene experience and nature connectedness was not significant (*β* = 0.063, *t* = 1.478, *p* = 0.140); *H5 was not supported*. Notably, this differentiated pattern held after the inclusion of control variables: the association of academic year with awe and of gender and nature-contact frequency with nature connectedness were small, and the focal paths were substantively unchanged, indicating that the results were robust to these demographic and exposure covariates.

### 4.6 Mediation analysis

The mediating roles of awe and nature connectedness were tested using bootstrap resampling with 5,000 replications, controlling for gender, academic year, and frequency of nature contact. Results are presented in Table 7.

**Table 7:** Mediation effects (bootstrap, 5,000 resamples, with controls).

Path	Effect	BootSE	95% LLCI	95% ULCI	Decision
Direct: CLSE → PEB	0.491	0.022	0.447	0.535	Significant
Indirect: CLSE → AW → PEB	0.361	0.036	0.290	0.432	Significant
Indirect: CLSE → CN → PEB	0.005	0.004	−0.002	0.014	Not significant
Chain: CLSE → AW → CN → PEB	0.040	0.013	0.017	0.068	Significant
Total: CLSE → PEB	0.897	0.017	0.864	0.930	Significant

LLCI and ULCI denote the lower and upper limits of the 95% percentile bootstrap confidence interval. Results with and without control variables were consistent.

The indirect association of campus landscape scene experience with pro-environmental behavior through awe was significant [Effect = 0.361, 95% CI = (0.290, 0.432)], supporting *H4*. The indirect association through nature connectedness alone was not significant [Effect = 0.005, 95% CI = (−0.002, 0.014)]; *H7 was not supported.* The chain indirect association CLSE → AW → CN → PEB was significant [Effect = 0.040, 95% CI = (0.017, 0.068)], supporting *H9*. These conclusions were consistent across models with and without control variables.

Overall, campus landscape scene experience was associated with pro-environmental behavior both directly and indirectly through awe and through the awe → nature connectedness chain, whereas the independent mediating role of nature connectedness was not supported. A summary of hypothesis testing results is presented in Table 8.

**Table 8:** Summary of hypothesis testing results.

No.	Hypothesis	Result
H1	Campus landscape scene experience → pro-environmental behavior	Supported
H2	Campus landscape scene experience → awe	Supported
H3	Awe → pro-environmental behavior	Supported
H4	Awe mediates CLSE → PEB	Supported
H5	Campus landscape scene experience → nature connectedness	Not supported
H6	Nature connectedness → pro-environmental behavior	Supported
H7	Nature connectedness mediates CLSE → PEB	Not supported
H8	Awe → nature connectedness	Supported
H9	Chain mediation: CLSE → AW → CN → PEB	Supported

## 5 Discussion

### 5.1 Main findings

Drawing on Attention Restoration Theory and self-transcendent emotion theory, this study constructed and tested a chain mediation model linking campus landscape scene experience to pro-environmental behavior through awe and nature connectedness. Three findings merit discussion in relation to the existing literature.

*First, campus landscape scene experience was positively associated with university students’ pro-environmental behavior.* This pattern is consistent with the restorative-environment literature (Berto, 2014; Zhao et al., 2019) and with campus-based studies reporting links between green space and student outcomes (Liu et al., 2024; Xu et al., 2024). It extends that work by locating the association in the everyday, embedded campus context rather than in dedicated nature-based travel or wilderness settings.

*Second, awe mediated the association between campus landscape scene experience and pro-environmental behavior.* This is consistent with prior evidence linking awe to ecological and prosocial outcomes (Piff et al., 2015; Yang et al., 2018; Yan et al., 2024; Liu et al., 2025). Beyond replicating the direction of this association, the present findings speak to its magnitude: the awe-mediated indirect effect (0.361) was considerably larger than the chain path through nature connectedness (0.040) and, descriptively, larger than the indirect effects reported in some tourism-based awe studies (e.g., Yang et al., 2018). A plausible reading is that the campus context, where awe-eliciting features are encountered repeatedly, may sustain the emotion differently than a single tourism episode; however, because effect sizes are not directly comparable across designs and samples, this comparison is offered as a hypothesis rather than a conclusion. We also consider an alternative, more cautionary explanation below in connection with construct overlap.

*Third, and most consequentially, nature connectedness did not independently mediate the association, yet operated as a necessary link within the awe → nature connectedness chain.* The direct CLSE → CN path was non-significant (*β* = 0.063, *p* = 0.140), the CLSE → CN → PEB indirect path was non-significant (95% CI includes zero), but the AW → CN path (*β* = 0.670) and the full chain CLSE → AW → CN → PEB [Effect = 0.040, 95% CI = (0.017, 0.068)] were both significant. This is the study’s most theoretically informative result, and it warrants a fuller explanation than the mere restatement of coefficients. We advance four, non-mutually-exclusive accounts. (a) Dispositional stability. Nature connectedness is typically conceptualized as a relatively stable, trait-like bond (Mayer and Frantz, 2004; Nisbet et al., 2009); a single perceptual appraisal of campus restorativeness may be too weak a signal to move a disposition that has formed over years of prior nature experience. (b) Emotional activation as a prerequisite. Consistent with self-transcendent emotion theory (Yaden et al., 2017; Stellar et al., 2017), the affective “jolt” of awe may be the proximal condition that renders restorative perception capable of updating the self–nature bond; without that activation, restorativeness alone does not translate into deeper connectedness. This aligns with Ng et al. (2023), who found awe to be the pathway through which nature learning related to prosocial responses. (c) A ceiling among already-connected students. Students who elect to spend time in a landscape-rich campus may already report high nature connectedness, compressing the variance available for campus perception to explain. (d) Multicollinearity and construct overlap. Given the high observed correlations among CLSE, awe, and PEB (Tables 3, 4), the strong CLSE → AW path may absorb variance that might otherwise appear on the CLSE → CN path, and part of the awe effect on behavior may reflect shared method or construct variance rather than a wholly distinct emotional process. We therefore interpret the awe pathway as robust in direction but potentially inflated in magnitude, and we treat the non-significant CLSE → CN association as substantively meaningful rather than as a null to be explained away. Collectively, these accounts favor an “emotionally activated” rather than “directly perceived” route to nature connectedness, extending prior antecedent models of connectedness (Lengieza and Swim, 2021) to the campus setting.

### 5.2 Theoretical contributions

This study offers three contributions. *First, it locates the environment–emotion–cognition–behavior sequence in an everyday, natural-cultural campus setting*, complementing a literature that has concentrated on tourism, wilderness, or dedicated outdoor programs (Yang et al., 2018; Yan et al., 2024; Liu et al., 2025). In doing so it responds to calls to examine routine, high-frequency nature contact. *Second, it integrates Attention Restoration Theory with self-transcendent emotion theory rather than invoking them separately*, and provides evidence for a differentiated internal structure of that integration: awe functions both as a direct mediator and as the entry point of the chain, whereas nature connectedness contributes only downstream of awe. This differentiation refines rather than merely confirms prior mediation accounts (Stellar et al., 2017; Lengieza and Swim, 2021). *Third, the non-significant direct CLSE → CN association is itself a theoretical contribution*: it qualifies the common assumption that nature exposure straightforwardly deepens connectedness (Capaldi et al., 2014), suggesting instead that self-transcendent emotional activation may be a boundary condition for that process. We advance this as a contribution to be tested, not as a settled claim, given the cross-sectional and single-site nature of the evidence.

### 5.3 Practical implications

The findings suggest three practical directions, offered as associational rather than prescriptive guidance.

*First, campus landscape design may benefit from a dual emphasis on restorativeness and awe-elicitation.* Beyond aesthetics and function, planners might incorporate features associated with awe—expansive water views, mature tree canopies, seasonal floral displays, and ecologically rich, biodiverse areas—so that spaces support both attention restoration and self-transcendent emotion.

*Second, campus natural landscapes may be productively integrated into ecological education.* Structured activities such as guided landscape walks or campus nature-observation modules could help convert incidental exposure into intentional engagement, within which awe and, in turn, nature connectedness may develop.

*Third, emotional activation deserves a place alongside knowledge transmission in pro-environmental education.* Because the present results suggest that exposure alone was not associated with deeper connectedness in the absence of awe, experiential methods that evoke awe—for example nature-based reflection or outdoor creative activities—may be a useful complement to information-centered approaches.

### 5.4 Limitations and future research

Three limitations qualify these conclusions and suggest directions for future work.

*First, the cross-sectional, self-report design does not permit causal inference.* The temporal ordering implied by the CLSE → AW → CN → PEB chain cannot be established from a single measurement occasion, and common-method influences, although mitigated by the ULMC check, cannot be fully excluded. The elevated CLSE–PEB and AW–PEB overlap reported in Table 4 further cautions against strong causal or magnitude claims for the awe pathway. Longitudinal, experimental, or experience-sampling designs, ideally with behavioral rather than self-reported outcomes, are needed to test the proposed ordering and to separate awe from adjacent constructs.

*Second, the single-university sample constrains generalizability.* Data came from one landscape-rich, AAA-rated scenic campus; the very feature that makes the site ecologically valid also bounds it. Multi-site studies spanning campuses that differ in landscape quality, institutional type, and regional and cultural context—including cross-cultural comparisons—are needed to assess how far the differentiated roles of awe and nature connectedness generalize.

*Third, campus landscape scene experience was measured as an aggregate via the PRS-11, and boundary conditions were not modeled.* Distinct landscape types—water bodies, woodlands, horticultural areas, and cultural features—may relate to awe and connectedness through different pathways. Future research could compare landscape categories, use virtual-reality or in-situ designs, test alternative model specifications (including reverse and non-mediated models), and introduce individual-level moderators (e.g., ecological values, nature sensitivity) and contextual moderators (e.g., season, weather) to build conditional models that clarify when the mechanism holds.

Fourth, the constructs exhibit substantial empirical overlap, which constrains the strength of the inferences that can be drawn from the structural estimates. As reported in Section 4.4, the HTMT values for the CLSE–PEB (0.926) and AW–PEB (0.931) pairs exceed the conventional 0.90 criterion, and although the bootstrapped confidence intervals excluded 1.00 and the alternative model comparisons indicated that combining these constructs significantly worsened model fit, discriminant validity cannot be regarded as established for these pairs. Consequently, the CLSE → PEB and AW → PEB coefficients, and the awe-mediated indirect effect in particular, should be interpreted as upper-bound estimates that may partly reflect shared construct or common-method variance rather than fully distinct psychological processes. The direction and relative ordering of the pathways—notably the contrast between the significant AW → CN path and the non-significant CLSE → CN path—are more robust than their absolute magnitudes. Future research should refine the measurement boundaries among environmental perception, self-transcendent emotion, and self-reported behavior, and should incorporate behavioral or observational outcome measures to reduce shared method variance.

## 6 Conclusion

Using survey data from 768 students at a landscape-rich Chinese university, this study examined how campus landscape scene experience relates to pro-environmental behavior through awe and nature connectedness. Campus landscape scene experience was positively associated with pro-environmental behavior both directly and indirectly. Awe served as a substantial mediator, and awe and nature connectedness formed a significant sequential chain, whereas nature connectedness did not mediate the association on its own—a pattern that points to awe as the emotional entry point through which restorative campus experience relates to a deeper self–nature bond.

Theoretically, the study integrates Attention Restoration Theory with self-transcendent emotion theory in an everyday campus context and identifies a differentiated internal structure in which nature connectedness operates downstream of awe rather than as an independent route. Practically, it suggests that campus landscape design and ecological education may be more effective when they cultivate awe alongside restorative exposure and knowledge, rather than relying on exposure alone. These conclusions are bounded by the study’s cross-sectional design, single-site sample, and the elevated overlap among several constructs; longitudinal, multi-site, and experimental research is needed to test the proposed sequence, examine boundary conditions, and establish causal direction. Within these limits, the findings offer an empirically grounded, theoretically integrated account of how routine campus nature experience may relate to young people’s pro-environmental behavior.

## Data Availability

The original contributions presented in the study are included in the article/supplementary material, further inquiries can be directed to the corresponding author.
